# The activity patterns of nonworking and working sled dogs

**DOI:** 10.1038/s41598-022-11635-5

**Published:** 2022-05-14

**Authors:** Ming Fei Li, Lavania Nagendran, Lauren Schroeder, David R. Samson

**Affiliations:** 1 Department of Anthropology, University of Toronto, 19 Ursula Franklin Street, Toronto, ON M5S 2S2 Canada; 2 Department of Anthropology, University of Toronto Mississauga, 3359 Mississauga Road, Mississauga, ON L5L 1C6 Canada

**Keywords:** Biological anthropology, Animal behaviour

## Abstract

There are limited studies investigating the combined effects of biological, environmental, and human factors on the activity of the domestic dog. Sled dogs offer a unique opportunity to examine these factors due to their close relationship with handlers and exposure to the outdoors. Here, we used accelerometers to measure the activity of 52 sled dogs over 30 days from two locations in Canada. The two locations differ in the working demands of dogs, therefore we used linear mixed effects models to assess how different factors impact daytime and nighttime activity of working versus nonworking dogs. During the daytime, we found that males were more active than females among nonworking dogs and younger dogs were more active than older dogs among working dogs. Alaskan huskies had higher activity levels than non-Alaskan husky breeds in working sled dogs during the day. Nonworking dogs were slightly more active during colder weather, but temperature had no effect on working dogs’ activity. The strongest predictor of daytime activity in working dogs was work schedule. These results indicate that the influence of biological factors on activity varied depending on dogs’ physical demands and human activity was the most powerful driver of activity in working dogs.

## Introduction

Sled dogs (*Canis familiaris*) have played an important role in the transportation of human populations in the Arctic and Subarctic^1^. Genetic evidence suggests that dogs commonly used in sledding (Greenland sled dogs, Siberian and Alaskan huskies, and Alaskan malamutes) are descended from an ancient lineage of dogs from Zokhov Island, Siberia dating back to 9500 years ago^2^. While advancements in transportation technology over the past century have changed, humans have shifted sled dogs’ roles to become more recreational (e.g., sled dog tours^3^) and commercial.

The nature of sled dog work provides a unique opportunity to examine activity patterns in this species. Sled dogs are more exposed to environmental conditions than companion dogs but are also heavily reliant on their human handlers. As such, they are a great model for exploring how human influences interact with biological as well as environmental factors that drive dog activity patterns.

Although studies have investigated the effects of biological variables (age, sex, and weight) on dog activity, findings were not always consistent. Several studies^4–6^ found younger dogs to display greater activity than older dogs. Females exhibited periods of heightened activity compared to males in companion dogs^5^, but males displayed higher activity than females during the night for sled dogs^7^. Weight also affected activity levels, with lighter dogs found to be more active than heavier dogs^6,8^. Conversely, Woods et al.^5^ found no significant effect of weight on dog activity. It is important to note that these studies included dogs of multiple breeds, and there is evidence that activity level of dogs may vary across breeds^9^. On the other hand, a study comparing the activity patterns of shelter dogs and owned dogs showed no significant differences in activity across age, sex, or weight^10^. Griss et al.^6^ also found no significant influence of sex on activity in dogs. One biological variable that is rarely considered in research on dog activity is the effect of neutering. In addition to controlling reproduction, neutering is often used to eliminate or decrease undesirable behaviors^11,12^. Increased bodyweight, increased appetite, and decreased activity were observed as long-term effects in dogs after neutering^13^. Previous results demonstrate that activity decreases with neutering^6^ and this trend is observed more commonly in males than females^14^.

The activity pattern of dogs across the 24-h period can be described as generally inactive, with periods of heightened activity^5–7,15^. Overall, dogs are considered diurnal and show more activity during the day than night^4,5,10,15,16^. Studies on drug-detection dogs demonstrated they were able to quickly recover from schedule changes due to their short sleep–wake cycle^17,18^. This suggests that daytime activity demands should not significantly impact the nighttime activity of dogs.

In addition to biological influences on dog activity, individuals who spend a considerable amount of time outdoors may be impacted by environmental conditions. First, activity may change in response to temperature. A previous study on sled dogs did not find any correlation between activity levels and temperature, but the observation period for that study did not include winter months^7^. Studies on pariah dogs in West Bengal^19^ and village dogs near Colola beach in Mexico^20^ found that dogs were less active at higher temperatures. This trend was also seen at higher latitudes in closely related species, like wolves (*Canis lupus*), that displayed a drop in activity once temperature rose above 20 °C^21^. Second, the nighttime activity of canids may fluctuate with the lunar cycle as light can influence predator feeding patterns, as well as prey behavior^22^. Higher activity levels on more moonlit nights have been recorded for wolves^21^, African wild dogs^23^, and coyotes^24,25^. The opposite trend (i.e., lower activity on more moonlit nights) has been observed in wild maned wolves^26^ and black-backed jackals^27^. Research on jackals suggest that the activity response to moonlight is variable and dependent on resource availability^28^. This is further supported by the more diurnal activity pattern of captive wolves who do not need to hunt for food^29^. Third, the housing condition of dogs can influence their activity patterns. Group-housed dogs exhibited greater activity than dogs that were housed individually^30^. The number of kennel mates may influence the activity patterns of dogs, especially at night when dogs are more restricted to their housing condition. In summary, existing evidence show that the activity level of sled dogs may decrease with increased temperatures and increase with more kennel mates, but no research has looked at how lunar cycle affects dog activity.

Dogs were the first domesticates, and have a shared history with our own species spanning back to at least 15,000 years ago^31,32^. Desired morphology, physiology, and behavior have been generated through artificial selection^33^. Kerepsi et al.^34^ found high levels of behavioral coordination between dogs and their owners during cooperative interactions. Multiple studies have shown that dog activity is heavily influenced by human presence and behavior^5–7,10,15,16,35^. Higher activity levels during the weekend compared to weekdays is likely a response to greater interaction with owners^5,35^. This trend was visible in companion dogs who reside with their owners, and also in free-ranging and working dogs^6,16^. The activity of free-ranging dogs in West Bengal, India was highest during times when humans were most active^16^. This is expected since free-ranging dogs in India have been observed to rely on human-derived foods^36^. Activity in guard dogs was reactive to external stimuli with barking correlated mainly with human and dog activity^15^. Higher levels of activity in sled dogs were also associated with human movement^7^. Griss et al.^6^ recently compared the activity patterns of free-ranging dogs to farm and family dogs. They found that dogs who were more independent from humans expressed a bimodal activity pattern which was not always observed in family dogs. The activity pattern of family dogs was more correlated with owner activity. These studies highlight the adaptability in the activity patterns of dogs as a response to human influence.

As past studies have demonstrated, dog activity is affected by a multitude of biological, environmental, and human factors (Supplemental Table S1), however few studies have evaluated the combined effects of these factors. In this context, we examine the activity data of outdoor-housed sled dogs from two separate locations in Canada. One of the sled dog facilities is located in Haliburton, Ontario and the other is in Canmore, Alberta. Haliburton sled dogs did not work during the study period; therefore, they represented nonworking dogs (Table 1). Canmore sled dogs represented working dogs (Table 2). We collected activity data using CamNTech MotionWatch 8 accelerometers placed on the inner collars of sled dogs for a period of 30 days. The aim of our study was to quantify the activity levels of working and nonworking sled dogs in order to answer the question: What are the effects of biological, environmental, and human variables on sled dog activity? Past findings mentioned above indicate that the activity patterns of companion and working dogs, and to a degree, free-ranging dogs, seem to be entrained to human activity. Therefore, in working sled dogs, we predicted human-mediated influences (work intensity/schedule, day type) would have larger effects on dog activity than biological (age, sex, weight, intactness, breed) and environmental (temperature, moon illumination) factors. In nonworking sled dogs, we predicted biological and environmental factors would have larger effects compared to human influences.

**Table 1:** Summary of multivariable approach using linear mixed-effects models in 29 Haliburton dogs.

Variable	Daytime model 1^a^			Nighttime model 1^b^		
	β	95% CI (low, high)	P	β	95% CI (low, high)	P
**Day type**						
Weekday (ref)	–	–	–	–	–	–
Weekend	0.027	− 0.037, 0.091	0.405	− 0.013	− 0.067, 0.0413	0.647
**Sex**						
Female (ref)	–	–	–	–	–	–
Male	**0.702**	**0.164, 1.240**	**0.031**	− 0.039	− 0.770, 0.692	0.926
Age	− 0.131	− 0.346, 0.085	0.297	− 0.256	− 0.548, 0.038	0.139
Weight	− 0.188	− 0.4212, 0.046	0.169	0.097	− 0.221, 0.414	0.596
**Intact**						
No (ref)	–	–	–	–	–	–
Yes	0.212	− 0.403, 0.827	0.550	0.542	− 0.294, 1.378	0.264
**Kennel**						
Two roommates (ref)	–	–	–	–	–	–
Three roommates	− 0.063	− 0.505, 0.378	0.803	0.248	− 0.352, 0.849	0.473
Temperature	− **0.055**	− **0.089,** − **0.020**	**0.002**	− **0.041**	− **0.065,** − **0.017**	**0.001**
Moon illumination	0.010	− 0.021, 0.041	0.543	0.022	− 0.004, 0.048	0.100
Sex × intact	− 0.562	− 1.263, 0.139	0.171	− 0.342	− 1.294, 0.611	0.533

Daytime model analysis of MotionWatch activity counts from 6:00 a.m. to 8:59 p.m. while nighttime model includes analysis of MotionWatch activity counts from 9:00 p.m. to 5:59 a.m.

^a^Overall model fit compared to the null model: N = 870, *χ*^2^ = 30.66, P < 0.001. Marginal R^2^ = 0.221, conditional R^2^ = 0.479.

^b^Overall model fit compared to the null model: N = 870, *χ*^2^ = 19.98, P = 0.018. Marginal R^2^ = 0.122, conditional R^2^ = 0.622.

Significant effects (P < 0.05) are in bold.

**Table 2:** Summary of multivariable approach using linear mixed-effects models in 23 Canmore dogs.

Variable	Daytime model 2^a^			Nighttime model 2^b^		
	Β	95% CI (low, high)	P	β	95% CI (low, high)	P
**Day type**						
Weekday (ref)	–	–	–	–	–	–
Weekend	**0.244**	**0.168, 0.322**	** < 0.001**	0.018	–0.051, 0.087	0.608
**Work**						
No (ref)	–	–	–	–	–	–
Yes	**0.895**	**0.819, 0.969**	** < 0.001**	− 0.0003	− 0.068, 0.068	0.993
**Sex**						
Female (ref)	–	–	–	–	–	–
Male	0.367	0.070, 0.664	0.051	0.246	− 0.232, 0.724	0.393
Age	** − 0.186**	** − 0.298, − 0.074**	**0.012**	0.011	− 0.169, 0.191	0.918
Weight	− 0.099	− 0.208, 0.010	0.142	− 0.055	− 0.231, 0.121	0.599
**Intact**						
No (ref)	–	–	–	–	–	–
Yes	0.340	0.062, 0.619	0.053	0.300	− 0.148, − 0.748	0.270
**Breed**						
Alaskan husky (ref)	–	–	–	–	–	–
Non-Alaskan husky	** − 0.454**	** − 0.694, − 0.215**	**0.005**	− 0.347	− 0.733, 0.038	0.144
Sex × intact	− 0.134	− 0.548, 0.280	0.588	− 0.199	− 0.865, 0.467	0.617
Temperature	− 0.008	− 0.046, 0.030	0.678	− 0.022	− 0.055, 0.011	0.189
Moon illumination	0.037	− 0.0001, 0.075	0.052	** − 0.036**	** − 0.070, − 0.003**	**0.033**

Daytime model analysis of MotionWatch activity counts from 6:00 a.m. to 8:59 p.m. while nighttime model includes analysis of MotionWatch activity counts from 9:00 p.m. to 5:59 a.m.

^a^Overall model fit compared to the null model: N = 690, *χ*^2^ = 561.5, P < 0.001. Marginal R^2^ = 0.598, conditional R^2^ = 0.684.

^b^Overall model fit compared to the null model: N = 690, *χ*^2^ = 15.07, P = 0.130. Marginal R^2^ = 0.152, conditional R^2^ = 0.562.

Significant effects (P < 0.05) are in bold.

## Results

In our analyses, we included individuals over the age of two, since this is when dogs have reached adulthood^37^. We only included individuals who were still working (i.e., not retired). We report results for the 52 individuals who met our inclusion criteria. These were 25 females and 27 males, 29 dogs were from Haliburton and 23 from Canmore. The mean age was 5.33 years old (± SD 2.46) and mean weight was 26.09 kg (± SD 4.58). All sled dogs at the Haliburton location were Alaskan huskies, while dogs from Canmore varied across husky breeds (Supplementary Table S2). At Haliburton, the mean temperature during the daytime was − 4.90 °C (± SD 3.76, range − 13.59 to 1.43) and during the nighttime was − 6.01 °C (± SD 4.42, range − 15.5 to 1.30), and the mean proportion of moon illumination was 0.53 (± SD 0.36, range 0 to 1). At Canmore, the mean temperature during the daytime was 4.51 °C (± SD 4.01, range − 10.86 to 2.31) and during the nighttime was − 5.71 °C (± SD 4.10, range − 12.60 to 1.19), and the mean proportion of moon illumination was 0.53 (± SD 0.36, range 0.002 to 0.999).

The response variables of interest in our analyses were daytime and nighttime activity, which were expressed as the sum of all 1-min MotionWatch (MW) activity counts over the daytime or nighttime period (see “Materials and methods” section). The mean daytime activity was 160,065 (± SD 94,176; median: 146,295; range 28,633 to 517,041) MW counts in Haliburton and 264,995 (± SD 187,356; median: 218,821; range 22,675 to 885,358) MW counts in Canmore dogs. The mean nighttime activity was 5382 (± SD 3382; median: 4871; range 508 to 30,279) MW counts in Haliburton and 9208 (± SD 7372; median: 7298; range 849 to 70,009) MW counts in Canmore dogs. We performed two sets of linear mixed-effects models, one for each location, to evaluate the effects of human-mediated influences, as well as biological and environmental variables on daytime and nighttime activity.

### Haliburton dogs

In Daytime model 1, we found sex and temperature to have significant effects on Haliburton dogs’ activity (Table 1). We found that males were more active than females (β = 0.702, P = 0.031) and dogs were less active during warmer days (β =  − 0.055, P = 0.002). In Nighttime model 1, we found that only temperature had a significant effect on Haliburton dogs’ activity (Table 1). Dogs were less active during warmer nights (β =  − 0.041, P = 0.001).

### Canmore dogs

**Figure 1:**
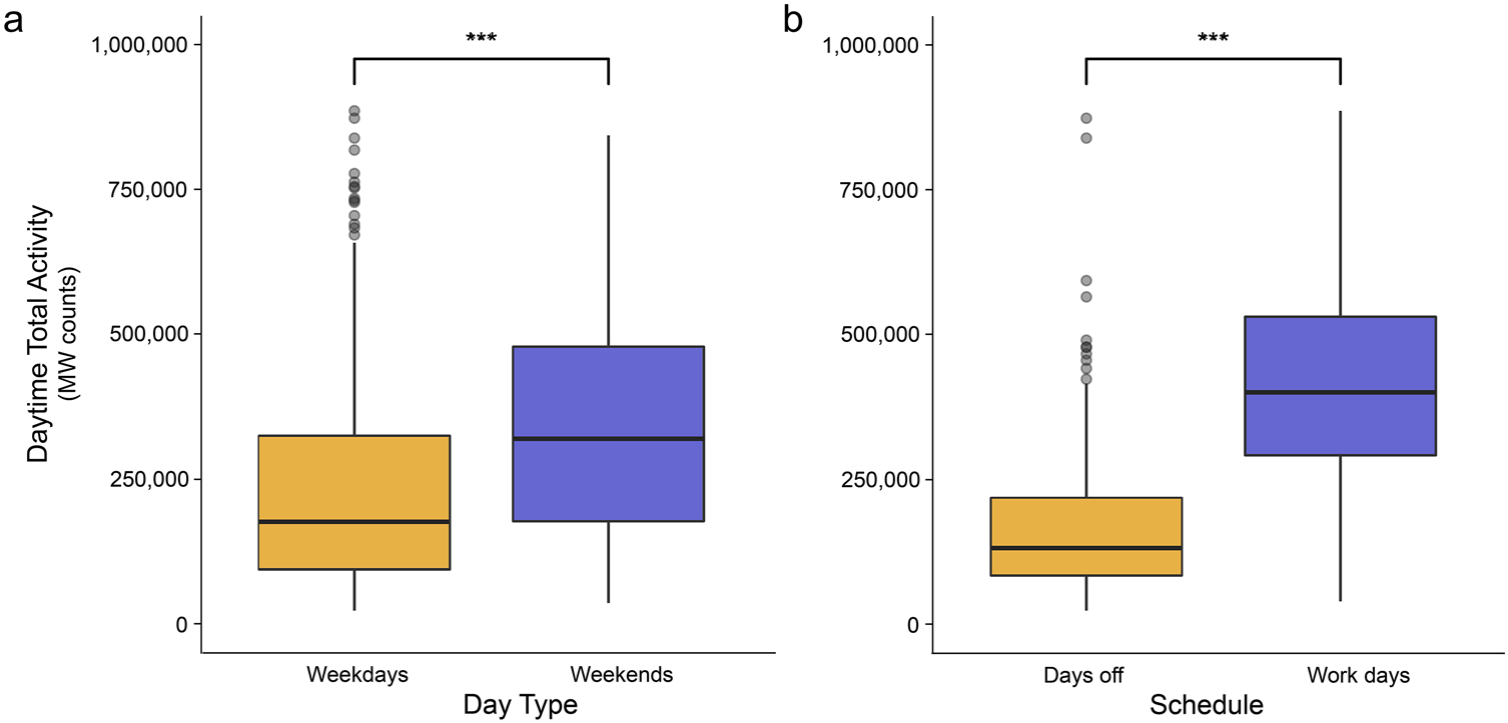
Box plot comparing the daytime activity of Canmore dogs on (**a**) weekdays and weekends, and (**b**) workdays and days off. Asterisks (***) indicate significant (P < 0.05) difference between groups.

**Figure 2:**
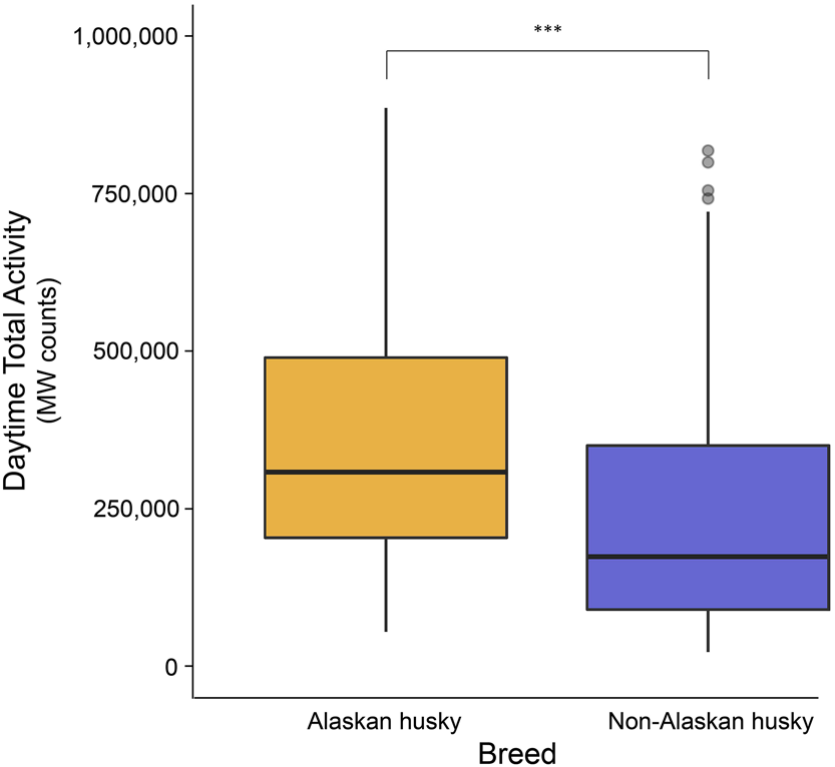
Box plot comparing the daytime activity of Alaskan husky (n = 6) versus non-Alaskan husky (n = 17) dogs from Canmore. Asterisks (***) indicate significant (P < 0.05) difference between groups.

In Daytime model 2, we found day type, work, age, and breed to have significant effects on Canmore dogs’ activity (Table 2). Dogs were more active during the day on weekends compared to weekdays (β = 0.244, P < 0.001; Fig. 1a) and were more active on work days than days off (β = 0.895, P < 0.001; Fig. 1b). Older dogs were less active than younger dogs (β =  − 0.186, P = 0.012) and non-Alaskan husky breeds were less active than Alaskan huskies (β =  − 0.454, P = 0.005; Fig. 2).

In Nighttime model 2, we found that only moon illumination had a significant effect on Canmore dogs’ activity (Table 2). Dogs were less active during nights with greater moon illumination (β =  − 0.036, P = 0.033). However, Nighttime model 2 was not significantly different from the null model, therefore we caution against interpreting this significant finding.

## Discussion

In this study, we examined how the daytime and nighttime activity of outdoor-housed working and nonworking sled dogs were affected by intrinsic and extrinsic factors. In line with our prediction, we found that working dogs’ activity intensity was mainly driven by human-induced factors like day type and work schedule. Among the biological variables we examined, sex had an effect on daytime activity in nonworking dogs, while age and breed affected activity in working dogs. Of the environmental variables, temperature had an effect on nonworking dogs; activity increased during colder temperatures. Taken together, our findings show that working sled dogs’ activity were more entrained to human activity than nonworking sled dogs. Additionally, biological factors had a stronger influence on activity than environmental conditions.

### Human influences

Both Haliburton and Canmore dogs are heavily reliant on handlers and therefore we expected the activity of sled dogs to be heavily influenced by human activity and behavior, which is a pattern observed across multiple studies examining human influence on companion, working, and free-ranging dogs^5–7,10,15,16,35^. Daytime model 2 (Canmore) showed that work schedule had the largest effect out of all the predictor variables. In other words, whether dogs worked or not explained the most variation in their daytime activity. When examining the influence of day type on the Daytime models, we found that dogs at Haliburton showed no significant differences in activity during the weekdays compared to weekends but Canmore dogs showed more activity during weekends. Canmore dogs likely expressed this pattern due to more tour bookings during the weekend since visitor availability is greatly constrained by the Monday to Friday work week. As a result of the COVID-19 lockdown in Ontario, the Haliburton dogs would not have experienced the difference in visitation between weekdays and weekends since visitations were restricted to the public during the study period. Research on companion dogs show that the “weekend effect” was also present with more activity expressed by dogs during weekends when owners were home^5,35,38^.

While our study examined the influence of day type and work schedule on sled dog activity, there are a number of additional human related factors that can also influence dog activity. These factors may include feeding time or handler presence in the dog enclosure. However, these variables differ from day to day, and thus were outside the scope of our research question. Future studies should consider these variables for a more detailed assessment of how specific human activity or behaviors influence dog activity. In addition, selective breeding by humans for performance traits in dogs should be factored in when possible.

### Biological variables

Out of the five biological variables we examined, sex significantly influenced daytime activity in nonworking Haliburton dogs and age significantly influenced daytime activity in working Canmore dogs. A recent study by Woods et al.^5^ found that female companion dogs were more active than males during the day, however they did not control for differences due to breed. Among Haliburton dogs, males were more active than females, which corroborates previous results from sled dogs^7^. However, no significant sex differences were observed in Canmore dogs. This suggests that working demands may reduce the variation observed in activity due to sex. We had predicted that intact dogs would be more active than neutered dogs based on owner reports that neutering led to decreased roaming and restlessness behavior in companion dogs^11,13^. On the contrary, we did not detect differences in activity between intact and neutered individuals for male and female sled dogs. While the specific hormonal pathways affecting the motivation toward, and sustainment of, physical activity in dogs remain unclear, reductions in testosterone and estrogen have been shown to impair physical activity in rodents^39^. Further research is needed to clarify whether removal of sex hormones through spaying and castration decreases activity in neutered individuals, particularly in dogs partaking in prolonged, high-intensity activity.

When examining the influence of age, we found no significant differences in the activity levels of dogs in the daytime and nighttime models for Haliburton, but age did influence the activity of sled dogs from Canmore during the daytime. Many studies show a decline in daytime locomotor activity associated with ageing^4,37,40^. Although Zanghi et al.^4,37^ observed age-related changes associated with nighttime activity, this was not observed in Siwak et al.^40^ or in nonworking sled dogs in our study. The lack of significant activity differences due to age in Haliburton dogs could be due to sampling bias since we only included adult dogs in this study, therefore our results do not reflect young or retired senior dog activity. On the other hand, Canmore dogs showed a decrease in activity with age in the daytime model which suggests that the influence of age on activity, even among healthy working dogs, is noticeable when dogs engage in high intensity activity. Few studies have examined the effect of weight on the activity patterns of healthy weight dogs. Existing research vary in results with Jones et al.^8^ and Griss et al.^6^ showing a negative relationship between weight and activity while Hoffman et al.^10^ and Woods et al.^5^ found no significant effect. In this study, we found no significant effect of weight on the activity of sled dogs from both locations.

Canmore dogs consisted of several different breeds, such as Siberian husky, Seppala husky, and Alaskan malamute. While the aforementioned breeds are all common sled dog breeds, our daytime model found that Alaskan huskies were significantly more active than non-Alaskan husky breeds. Alaskan huskies are the result of selectively breeding among several working dog breeds to achieve the desired traits (e.g., speed, endurance, work ethic) for sled dog racing^41^. Staff at Canmore confirmed that Alaskan huskies were the hardest working breed and ideal for running sled tours due to their strong desire to pull for long durations (J. Arsenault, personal communication). Alaskan huskies may have been preferentially chosen to pull longer tours, therefore, leading to higher activity levels detected by the accelerometers. As such, the difference in activity between breeds could be a combination of biological (i.e., physiological) and human (i.e., staff preference) factors.

### Environmental variables

Overall, environmental factors had the smallest effect on sled dog activity compared to biological and human variables. Domesticated animals (dogs) are expected to be less attentive to their environment compared to wild species (wolves) because domesticates rely less on external conditions for hunting, mating, and survival^42^. Our results showed that temperature and moon illumination had significant, albeit small, effects on sled dog activity. We found that Haliburton dogs exhibited more activity during colder temperatures than warmer temperatures. However, Canmore dogs did not express significant differences in activity with changes in temperature. The National Research Council reported the lower critical temperature for Siberian huskies to be 0 °C^43^, but we believe the lower critical temperature for sled dogs is substantially lower than 0 °C because these dogs spend all of their time outdoors and have developed greater tolerance for cold winters than companion counterparts^44^. Temperatures at Haliburton were on average lower than Canmore, so the higher activity in Haliburton dogs could indicate the onset of nighttime shivering, huddling, and burrowing behaviors as temperatures at Haliburton approached or surpassed their lower critical threshold. Canmore dogs were also fed later in the day and had a more high-fat diet compared to Haliburton dogs. In addition, it is plausible that working sled dogs are more tolerant to temperature fluctuations. More research is needed to elucidate the physiological mechanisms associated with diet and physical activity that may underlie dogs’ tolerance to cold temperature.

Another environmental variable that is known to affect foraging and predation patterns of animals is moonlight^22^. Activity patterns relative to moonlight is shown to be variable in some canid species and is dependent on resource availability^28^. While wild wolves expressed greater activity on moonlit nights^21^, captive wolves showed a more diurnal activity pattern^29^. Sled dogs are provisioned and do not rely on predation for food, so we did not expect moonlight to affect their activity. Contrary to our prediction, sled dogs from Canmore expressed less activity levels in the nighttime model when there was more moonlight. It is important to note that the overall nighttime model for Canmore was not significant, so results should be interpreted with caution. The observed pattern may be a response to heightened wildlife activity during moonlit nights since the study location is surrounded by expansive forested environments. Dogs have been selectively bred for a number of roles such as hunting, guarding, and herding^33,45^ which require high attentiveness to environmental stimuli^15,18^. On the other hand, Haliburton dogs did not show any significant trends in activity levels associate with moonlight. The effects of moonlight may not be as strong in Haliburton dogs since these dogs are kept in their grouped kennels (Supplementary Fig. S1a), which would reduce their exposure to moonlight compared to Canmore dogs who can roam out of their covered housing as they please. Future studies should record ambient noise to assess whether the activity pattern of dogs is correlated to environmental sounds or the sounds of neighboring dogs.

The effect of number of kennel mates on activity was modelled for Haliburton dogs which were kept in groups of two or three during the night and parts of the day. Hubrecht et al.^30^ found that group-housed dogs expressed more activity than solitary dogs. Our study found that the number of dogs in each group did not influence dog activity. We were not able to compare group housed dogs to solitary dogs because the Haliburton dogs included in this study did not have a kennel to themselves. Although each kennel houses two or three dogs, the kennels are proximate to one another (Supplementary Fig. S1a) which may still allow neighboring dog activity to influence individual dog activity.

### Nighttime activity

Interestingly, Nighttime model 2, which only included Canmore dogs, was not significantly different from the null model, which means none of the predictors were notable drivers of differences in nighttime activity among working sled dogs. Woods et al.^5^ also found that none of the variables influencing daytime activity (sex, age, day type) had an effect on nighttime activity in companion dogs. An explanation for the lack of significant effects on nighttime activity in working sled dogs is their sleep–wake pattern. Dogs are polyphasic sleepers (i.e., have multiple sleep bouts)^4^ and Adams and Johnson^17^ found that dogs had on average 23 sleep bouts lasting 21 min each over the course of 8 h. These short sleep cycles in dogs may be key for quick recovery from schedule changes. In drug–detector dogs, it was found that dogs only experienced a “first-night” effect of disrupted sleep if they were returning from an extended break (i.e., several weeks), and their sleep–wake cycles resumed normalcy after the first night^46^. Working sled dogs from Canmore maintained a consistent work schedule during the study duration without any prolonged breaks; in fact, all dogs undergo training a month prior to the busy season. Dogs’ polyphasic sleep pattern (i.e., multiple sleep bouts throughout the 24 h period), coupled with the short duration of sleep–wake cycles, likely underlie their ability to quickly adapt to changing daytime schedules^17,46^.

## Conclusions

The comparison of working and nonworking sled dogs showed that environmental conditions, like temperature and moonlight, had relatively minor effects on dog activity despite sled dogs being outside all day. Biological factors, such as sex and age, had different effects on activity depending on dogs’ physical demands. Overall, work demands mediated by human schedule was the strongest driver of differences in activity of working sled dogs. While the current study focused on the intensity of activity, a future study could include variation in the patterning of daily activity (i.e., when dogs are active during the 24 h period), which could provide further insight into why we failed to detect any significant drivers of nighttime activity in working sled dogs. While most studies conclude that dogs are diurnal^4,10,15,16^, there is evidence that dogs exhibit bimodal activity peaks and may actually be cathemeral (i.e., active during the day and night)^5,7^. It is plausible that *C. familiaris* may be a facultative cathemeral species that have adapted to match humans’ more diurnal activity pattern. Finally, we demonstrate that the use of non-invasive technology, such as accelerometry, can facilitate research on dog activity with minimal interruption to their regular daily tasks, which is very useful when studying working dogs.

## Materials and methods

### Ethics statement

This study was approved by the University of Toronto Animal Care Committee (Protocol # 20012651) and all methods were performed in accordance with relevant guidelines and regulations. Researchers did not interfere with the housing, diet, or management of the dogs. Methods were also approved by staff at Haliburton Forest and Wild Life Reserve and Snowy Owl Sled Dog Tours. This study complies with the ARRIVE (Animal Research: Reporting of In Vivo Experiments) guidelines^47^.

### Study site and subjects

We recruited 60 sled dogs (29 females, 31 males) from two facilities in Canada: 30 from Haliburton Forest and Wild Life Reserve (located in Haliburton, Ontario; 45.22° N, 78.59° W) and 30 from Snowy Owl Sled Dog Tours (located in Canmore, Alberta; 51.09° N, 115.36° W). These locations will be referred to as Haliburton and Canmore, respectively, throughout the paper. At Haliburton, dogs were housed in sex-specific outdoor enclosures (49.6 m long and 12.2 m wide) with kennels measuring 1.5 m by 2 m on average. Kennel size varied depending on how many individuals were housed in the kennel, which ranged from one to three dogs (Supplemental Fig. S1). During the daytime, sled dogs from both Haliburton and Canmore were given time to roam free in their outdoor enclosures when they were not participating in the trail runs. The amount of time sled dogs were loose in their enclosure rather than restricted to their individual kennel varied day to day and by individual—this was dependent on a number of factors such as weather conditions and dog behavior. Dogs were fed high-performance kibble once per day, at 3:00 pm, and received water throughout the day. All Haliburton sled dogs were Alaskan huskies. At Canmore, dogs were also housed in sex-specific outdoor enclosures, measuring 8000 m^2^ in area (Supplemental Fig. S1). Each dog had their own house (length: 1 m, width: 0.7 m, height: 0.6 m), built for insulation during winter months. Dogs were tethered to their house and could roam up to 2.5 m around their house throughout the day. Dogs were fed high-performance kibble and diet was supplemented with meat. At Canmore, the first feeding was between 6:30 and 8:30 am, dogs received water and soup throughout the day, and the last feeding session was between 4:00 and 7:00 pm. There were multiple dog breeds at Canmore (e.g., Alaskan husky, Canadian Indian mix, and Seppala Siberian). At both locations, staff estimated dogs to be in contact with humans for a minimum of eight hours a day. We asked staff to provide information on dog breed, sex, weight, medical history, whether they were neutered, as well as working schedules, if applicable (see Supplemental Table S2 for dog signalment information).

### Data collection

We collected data between December 6, 2020 and January 19, 2021. We chose this period because this is the busiest part of the working season for sled dogs, thus, dogs would be the most active. Unfortunately, due to Ontario’s COVID-19 provincial lockdown, Haliburton cancelled all sled dog tour bookings during the study period. Handlers at Haliburton occasionally practised running routes with the dogs, but none of the dogs worked during the study. Canmore on the other hand, was able to continue with tours due to different provincial guidelines.

To record dog activity, we used the CamNtech MotionWatch 8 accelerometer to quantify movement. The accelerometer has a piezoelectric film that detects and records movements as acceleration waveforms over each second. Acceleration measurements are then processed by MotionWatch 8’s on-board software to produce MotionWatch (MW) counts, which represents a measure of activity for a predetermined time period. We set the accelerometer to record MW counts for 1-min epochs (i.e., each data point is the sum of MW counts over 60 s). Accelerometers have been previously used to measure dog activity (e.g.^5,10,48^).The MotionWatch 8 accelerometer measurements were: 36 mm (length), 28.2 mm (width), 9.4 mm (depth), and 9.1 g (weight). We attached the accelerometer to the inner side (i.e., side in contact with dog) of a nylon neck collar using Gorilla Tape and we made sure that only smooth, non-adhesive materials were in contact with the dog (see Supplemental Information Fig. S2). Similar attachment methods have been used by Hoffman et al.^10^. We prepared and shipped all equipment to the two locations and staff placed collars on the dogs—researchers did not interact with the dogs. At the end of the study duration, all equipment were shipped back to us, and we downloaded the raw activity count data using MotionWare (version 1.2.23).

### Environmental data

We obtained hourly Canmore temperature data from a weather station located 17.49 km from the study location (climate.weather.gc.ca). We collected hourly temperature data from Haliburton using a Kestrel 5400 Heat Stress Tracker placed next to the outdoor enclosure. For analyses, we calculated the average daytime and nighttime temperatures for each date. Moon illumination information was obtained from dateandtime.com and daily sunrise and sunset times were from sunrise-sunset.org. Moon illumination is the proportion of the moon’s visible surface that is illuminated by the sun when the moon passes the local meridian. Values range from 0 (no illumination during new moon) to ~ 1 (maximum illumination during full moon).

### Data analysis

For each individual, we included 30 days of data (December 12, 2020 to January 10, 2021 for Haliburton dogs and December 18, 2020 to January 16 for Canmore dogs). We first divided the 24 h period into “daytime”, which was from 6:00 am to 8:59 pm and “nighttime”, which was from 9:00 pm to 5:59 am. Daytime included the earliest possible wake-up times (i.e., earliest handler arrival at Canmore was 6:30 am) and 2 h after the last possible feeding session (i.e., 7:00 pm at Canmore).

To test our predictions, we evaluated the relative effects of human, biological, and environmental variables on working and nonworking sled dogs’ daytime and nighttime activity using a multivariable approach where we looked at all the fixed effects in the same linear mixed-effects model (LMM). In Daytime model 1 and Nighttime model 1, we only included data from Haliburton dogs. In Daytime model 2 and Nighttime model 2, we only included Canmore dogs. The response variable in the daytime models was the daytime total activity, which was the sum of all 1-min MW counts over one daytime period. The response variable in the nighttime models was the nighttime total activity, which was the sum of all 1-min MW counts over one nighttime period. To meet model assumptions, we used log transformation on the response variable since activity count was positively skewed^49^. In model 1, the fixed effects were day type (weekend/weekday), sex (male/female), age (continuous), weight (continuous), intact (yes/no), kennel (two/three roommates), temperature (continuous), and moon illumination (continuous). We also included an interaction effect for sex and intact because mating behaviors differ in males and females, so the intact condition could have different effects on activity in the two sexes. In model 2, we included all the fixed effects from model 1 except for kennel since Canmore dogs had individual houses. We also added breed (Alaskan husky/non-Alaskan husky) and work schedule (whether dogs had worked the day prior: yes/no) as additional fixed effects. We coded breed as a binary variable because the sample size for some of the non-Alaskan breeds were very small (e.g., 1 Alaskan malamute) and there were several mixed breeds (Supplemental Table S2). To allow for comparison of effect size across variables, all continuous variables were scaled by subtracting the mean and dividing by the standard deviation^50^. We set ID as a random effect since we had repeat observations (one each per day) for each individual.

For all LMMs, overall model significance was determined using a likelihood ratio test comparing the full model to a null model with only the random effect. We also reported the marginal and conditional R^2^ values for all LMMs^51^. We used the following R functions and packages: lmer() function in *lme4*^52^ for fitting LMMs, simulateResiduals() and plot() in *DHARMa*^53^ for model diagnostics, and r.squaredGLMM() in *MuMIn*^54^ for calculating marginal and conditional R^2^ values. All analyses were performed in R version 4.0.1 for Mac OS X^55^. All statistical tests were two-tailed with alpha set to 0.05.

## Supplementary Information

Supplementary Information.

## Data Availability

Data and codes used in this paper are available upon request from the corresponding authors.
