# Supplementary material for: The activity patterns of nonworking and working sled dogs
Source: Sci Rep. 2022 May 14;12:7999. doi: 10.1038/s41598-022-11635-5 (PMC9107014; doi:10.1038/s41598-022-11635-5)
Supplement: Supplementary file 1 — Supplementary Information. [file 41598_2022_11635_MOESM1_ESM.pdf]

Title: The activity patterns of nonworking and working sled dogs

Authors: Ming Fei Li[*, 1], Lavania Nagendran[*, 1], Lauren Schroeder[2], David R. Samson[2]

[*] Co-first authors
[1] Department of Anthropology, University of Toronto, 19 Ursula Franklin Street, Toronto ON, Canada M5S 2S2
[2] Department of Anthropology, University of Toronto Mississauga, 3359 Mississauga Road, Mississauga ON, Canada L5L 1C6

Corresponding authors:
Ming Fei Li (mf.li@mail.utoronto.ca)
Lavania Nagendran (lavania.nagendran@mail.utoronto.ca)

Supplemental Information

Table S1. Summary of predictions for the effects of biological, environmental, and human factors on daytime and nighttime activity in dogs and other canid species (> refers to "greater activity than").

Category	Variable	Daytime activity	Nighttime activity	Ref
Biological	Sex	No effect	Males > Females	1
	Age	Younger > Older		2–4
	Weight	No effect in healthy dogs		3,5
	Intact	Intact > Neutered		4,6,7
	Breed	Differs across breeds		8
Environmental	Temperature	Lower > higher temperatures		9,10
	Moon illumination	No effect		11
	Kennel	More > Fewer kennel mates		12
Human	Day type	Weekends > Weekdays		3,13
	Work schedule	Work > days off	No effect	14,15

Table S2. Signalment information for study participants.

Location	ID	Breed	Sex	Age (yr)	Weight (kg)	Intact	# Days Worked [a]
Haliburton	CAN001	Alaskan husky	F	8	22.22	Y	0
Haliburton	CAN002	Alaskan husky	M	2	24.94	Y	0
Haliburton	CAN003	Alaskan husky	M	2	22.68	Y	0
Haliburton	CAN004	Alaskan husky	M	2	20.41	Y	0
Haliburton	CAN005	Alaskan husky	F	2	18.14	N	0
Haliburton	CAN006	Alaskan husky	M	3	27.21	Y	0
Haliburton	CAN007	Alaskan husky	M	3	29.48	Y	0
Haliburton	CAN008	Alaskan husky	F	7	22.68	N	0
Haliburton	CAN009	Alaskan husky	M	4	31.75	N	0
Haliburton	CAN010	Alaskan husky	M	11	29.48	Y	0
Haliburton	CAN011	Alaskan husky	M	5	31.75	Y	0
Haliburton	CAN012	Alaskan husky	M	9	27.21	Y	0
Haliburton	CAN013	Alaskan husky	M	5	27.21	Y	0
Haliburton	CAN014	Alaskan husky	F	8	23.13	Y	0
Haliburton	CAN015	Alaskan husky	M	7	27.21	N	0
Haliburton	CAN017	Alaskan husky	M	3	27.21	Y	0
Haliburton	CAN018	Alaskan husky	M	2	25.85	Y	0
Haliburton	CAN019	Alaskan husky	M	4	28.57	Y	0
Haliburton	CAN020	Alaskan husky	F	6	23.58	N	0
Haliburton	CAN021	Alaskan husky	F	2	17.69	N	0
Haliburton	CAN022	Alaskan husky	M	4	27.21	Y	0
Haliburton	CAN023	Alaskan husky	F	7	27.21	N	0
Haliburton	CAN024	Alaskan husky	F	6	22.68	N	0

Haliburton	CAN025	Alaskan husky	F	6	22.68	N	0
Haliburton	CAN026	Alaskan husky	F	4	24.94	N	0
Haliburton	CAN027	Alaskan husky	F	10	27.21	N	0
Haliburton	CAN028	Alaskan husky	F	7	23.58	N	0
Haliburton	CAN029	Alaskan husky	F	4	24.49	N	0
Haliburton	CAN030	Alaskan husky	F	10	24.94	N	0
Canmore	CAN031	Alaskan husky	M	6	20.41	N	14
Canmore	CAN032	Siberian mix	M	2	31.75	Y	11
Canmore	CAN033	Canadian Indian mix	M	8	34.01	N	13
Canmore	CAN035	Canadian Indian mix	M	9	24.94	N	8
Canmore	CAN037	Alaskan husky	M	6	24.94	Y	14
Canmore	CAN038	Alaskan husky	M	5	34.01	N	13
Canmore	CAN039	Canadian Indian mix	M	4	31.75	Y	14
Canmore	CAN040	Seppala mix	M	3	29.48	N	13
Canmore	CAN041	Seppala mix	F	5	24.94	N	13
Canmore	CAN042	Alaskan husky	F	6	18.14	Y	12
Canmore	CAN043	Canadian Indian mix	F	4	27.21	N	14
Canmore	CAN044	Alaskan husky	F	7	22.68	Y	14
Canmore	CAN045	Siberian mix	F	2	22.68	Y	14
Canmore	CAN047	Seppala mix	F	8	24.94	N	10
Canmore	CAN048	Alaskan husky	M	7	27.21	N	12
Canmore	CAN049	Seppala mix	F	3	27.21	N	13
Canmore	CAN050	Canadian Indian mix	F	6	20.41	Y	14
Canmore	CAN051	Seppala mix	F	9	24.94	N	4
Canmore	CAN053	Alaskan malamute	M	4	43.08	Y	14
Canmore	CAN054	Canadian Indian mix	M	6	31.75	Y	11
Canmore	CAN055	Siberian mix	M	2	31.75	Y	11
Canmore	CAN056	Canadian Indian mix	F	8	27.21	Y	13
Canmore	CAN058	Canadian Indian mix	F	4	18.14	Y	14

[a] Out of the 30 days of data collection.

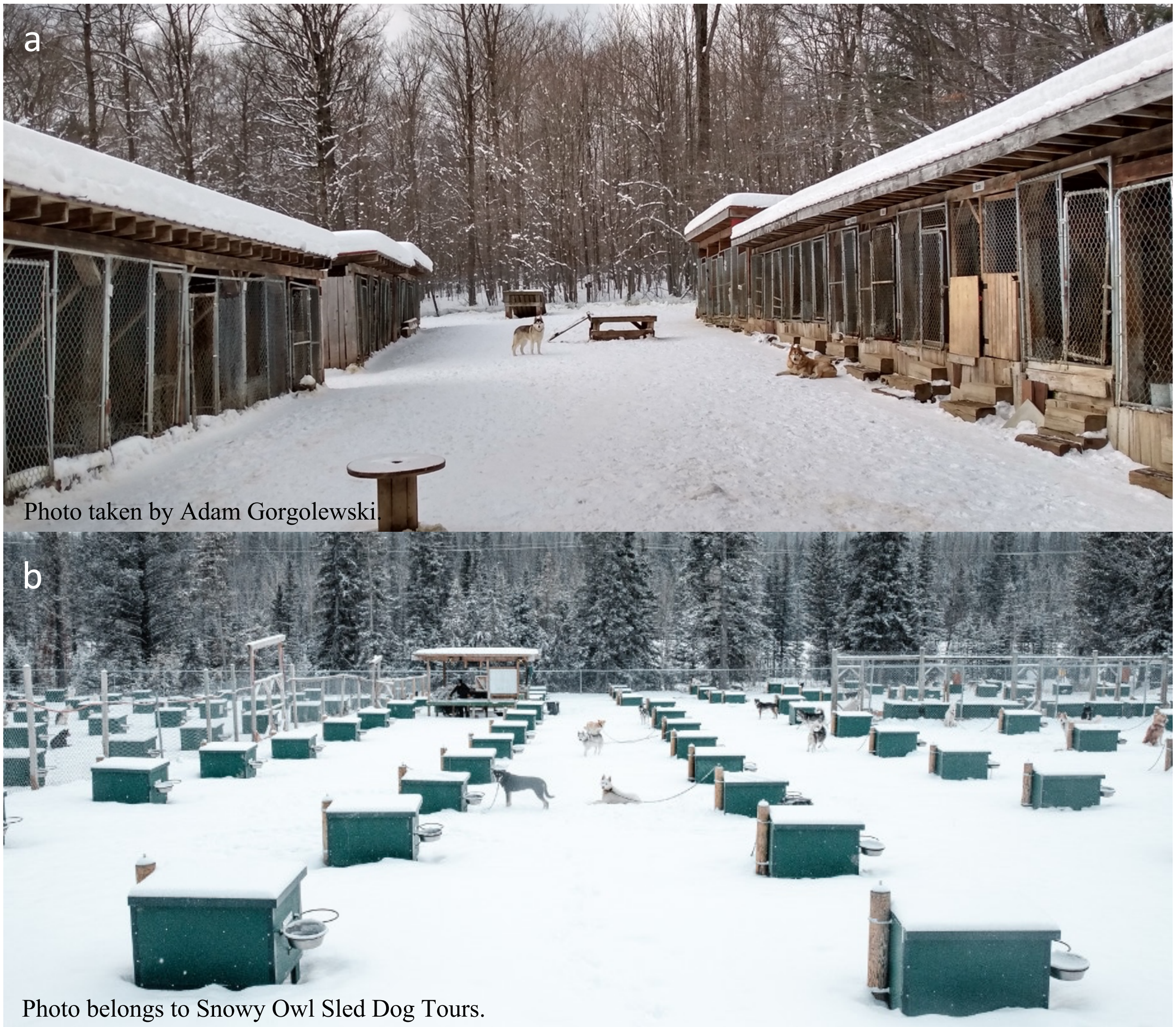

Figure S1. Images of sex-specific outdoor enclosure for (a) Haliburton and (b) Canmore.

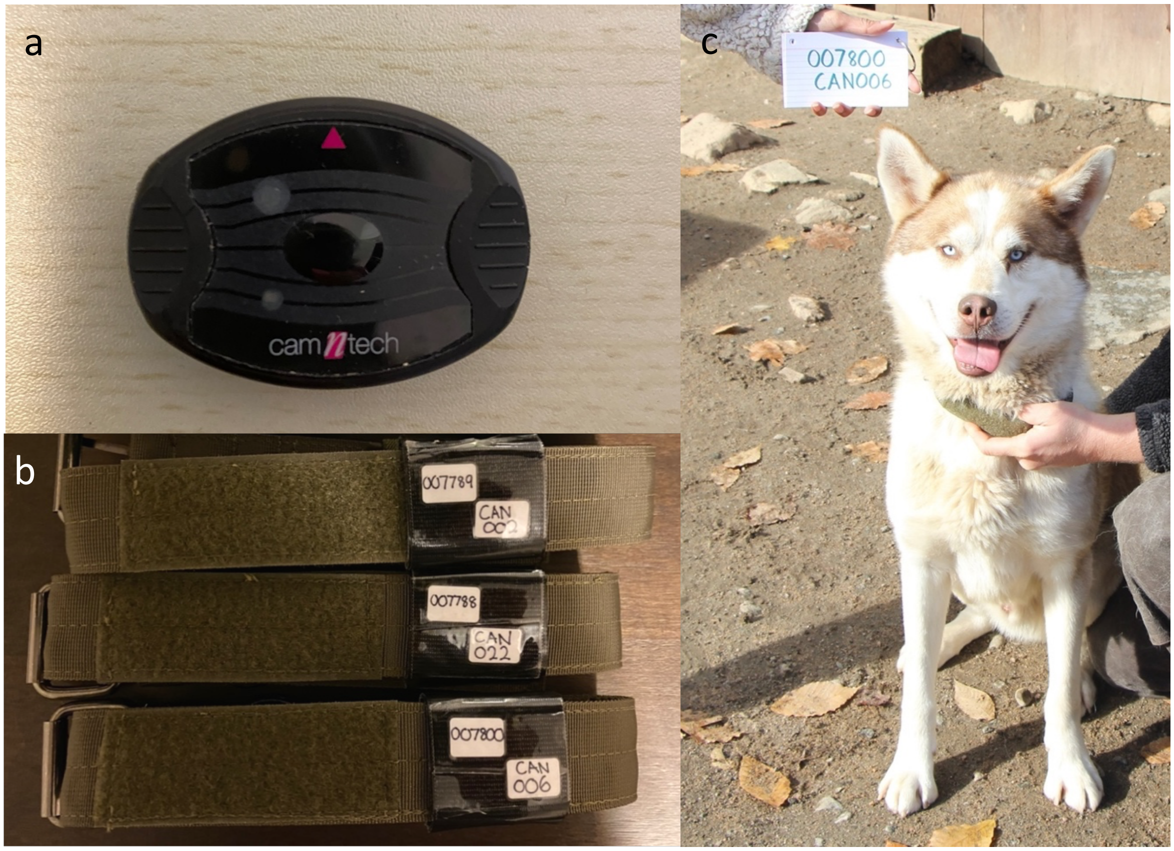

Figure S2. Images of (a) MotionWatch 8 accelerometer, (b) accelerometer attached to collar via Gorilla Tape, and (c) collar worn by a study participant.

References

1. Delude, L. A. Activity patterns and behaviour of sled dogs. *Applied Animal Behaviour Science* **15**, 161–168 (1986).
2. Zanghi, B. M., Kerr, W., de Rivera, C., Araujo, J. A. & Milgram, N. W. Effect of age and feeding schedule on diurnal rest/activity rhythms in dogs. *Journal of Veterinary Behavior* **7**, 339–347 (2012).
3. Woods, H. J. *et al.* A functional linear modeling approach to sleep–wake cycles in dogs. *Sci Rep* **10**, 22233 (2020).
4. Griss, S. *et al.* If they could choose: How would dogs spend their days? Activity patterns in four populations of domestic dogs. *Applied Animal Behaviour Science* 105449 (2021) doi:10.1016/j.applanim.2021.105449.
5. Hoffman, C. L., Ladha, C. & Wilcox, S. An actigraphy-based comparison of shelter dog and owned dog activity patterns. *Journal of Veterinary Behavior* **34**, 30–36 (2019).
6. Maarschalkerweerd, R. J., Endenburg, N., Kirpensteijn, J. & Knol, B. W. Influence of orchiectomy on canine behaviour. *Veterinary Record* **140**, 617–619 (1997).
7. Palestrini, C. *et al.* Influence of Gonadectomy on Canine Behavior. *Animals* **11**, 553 (2021).
8. Pickup, E., German, A. J., Blackwell, E., Evans, M. & Westgarth, C. Variation in activity levels amongst dogs of different breeds: results of a large online survey of dog owners from the UK. *J Nutr Sci* **6**, e10 (2017).
9. Oppenheimer, E. C. & Oppenheimer, J. R. Certain behavioral features in the pariah dog (Canis familiaris) in West Bengal. *Applied Animal Ethology* **2**, 81–92 (1975).
10. Theuerkauf, J. R. *et al.* Daily patterns and duration of wold activity in the Białowieża Forest, Poland. *Journal of Mammalogy* **84**, 12 (2003).
11. Rotem, G., Berger, H., King, R., Bar (Kutiel), P. & Saltz, D. The effect of anthropogenic resources on the space-use patterns of golden jackals. *The Journal of Wildlife Management* **75**, 132–136 (2011).
12. Hubrecht, R. C., Serpell, J. A. & Poole, T. B. Correlates of pen size and housing conditions on the behaviour of kennelled dogs. *Applied Animal Behaviour Science* **34**, 365–383 (1992).
13. Dow, C., Michel, K. E., Love, M. & Brown, D. C. Evaluation of optimal sampling interval for activity monitoring in companion dogs. *American Journal of Veterinary Research* **70**, 444–448 (2009).
14. Adams, G. J. & Johnson, K. G. Guard dogs: sleep, work and the behavioural responses to people and other stimuli. *Applied Animal Behaviour Science* **46**, 103–115 (1995).
15. Gerth, N., Redman, P., Speakman, J., Jackson, S. & Starck, J. M. Energy metabolism of Inuit sled dogs. *J Comp Physiol B* **180**, 577–589 (2010).
